# Gut-on-a-chip for exploring the transport mechanism of Hg(II)

**DOI:** 10.1038/s41378-022-00447-2

**Published:** 2023-01-01

**Authors:** Li Wang, Junlei Han, Weiguang Su, Anqing Li, Wenxian Zhang, Huimin Li, Huili Hu, Wei Song, Chonghai Xu, Jun Chen

**Affiliations:** 1 School of Mechanical Engineering, Qilu University of Technology (Shandong Academy of Sciences), Jinan, 250353 China; 2 Shandong Institute of Mechanical Design and Research, Jinan, 250353 China; 3 The Key Laboratory of Experimental Teratology, Ministry of Education and Department of Genetics, School of Basic Medical Sciences, Shandong University, 250012 Jinan, China; 4 The Research Center of Stem Cell and Regenerative Medicine, School of Basic Medical Sciences, Cheeloo Medical College, Shandong University, 250012 Jinan, China; 5 Department of Oncology, Shandong Provincial Hospital Affiliated to Shandong University, Jinan, Shandong 250021 China

**Keywords:** Engineering, Nanoscience and technology

## Abstract

Animal models and static cultures of intestinal epithelial cells are commonly used platforms for exploring mercury ion (Hg(II)) transport. However, they cannot reliably simulate the human intestinal microenvironment and monitor cellular physiology in situ; thus, the mechanism of Hg(II) transport in the human intestine is still unclear. Here, a gut-on-a-chip integrated with transepithelial electrical resistance (TEER) sensors and electrochemical sensors is proposed for dynamically simulating the formation of the physical intestinal barrier and monitoring the transport and absorption of Hg(II) in situ. The cellular microenvironment was recreated by applying fluid shear stress (0.02 dyne/cm^2^) and cyclic mechanical strain (1%, 0.15 Hz). Hg(II) absorption and physical damage to cells were simultaneously monitored by electrochemical and TEER sensors when intestinal epithelial cells were exposed to different concentrations of Hg(II) mixed in culture medium. Hg(II) absorption increased by 23.59% when tensile strain increased from 1% to 5%, and the corresponding expression of *Piezo1* and *DMT1* on the cell surface was upregulated.

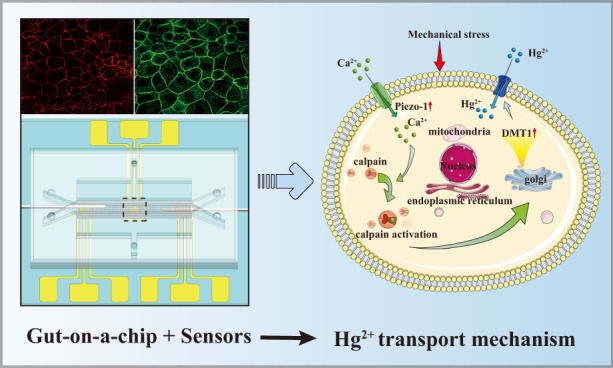

## Introduction

Nonbiodegradable mercury ions (Hg(II)) can accumulate in the body even at a low concentration (5 μM)^1^, which can damage the heart, kidney, intestine, and central nervous system^2–11^. At the subcellular level, Hg(II) interacts with antioxidant proteins, DNA repair enzymes, and proteins that maintain cell homeostasis, producing disordered cellular structure and function^12–14^. Hg(II) absorption mainly occurs in the small intestine. The ingestion of a high concentration of Hg(II) could cause intestinal bleeding and even perforation in a short time. Long-term intake of low concentrations of mercury ions may lead to chronic intestinal diseases (e.g., intestinal inflammation, mucosal injury and abnormal peristalsis). The intestinal epithelium is the first barrier following the ingestion of mercury. It can limit penetration of Hg(II) into the bloodstream and reduce the harmful effects on different target tissues. Thus, a reasonable intestinal model is of great significance to explore the transport mechanism of Hg(II) entering target cells and can efficiently provide comprehensive information for recognizing its toxicological effects.

In vivo animal models and Transwell models (static culture) are traditionally used for studying intestinal absorption and transport of Hg(II)^15–18^. Synthetic physiological phenomena (the signs and symptoms of poisoning in the gut, liver, kidney, and brain) after ingesting Hg(II) were investigated in vivo in a mouse model. Continuous infusion of high-dose Hg(II) (33.6 mg/kg) for 7 days caused mild intestinal inflammation, along with a 10% and 5% downregulation of peptide transporter-1 and Ost-β, respectively^18^. The accumulation of Hg(II) in the intestine was positively related to the expression of *DMT1* protein^19^. Due to species differences, animal models fail to faithfully represent human physiology and do not accurately reflect the human transport mechanism of Hg(II). In the Transwell model, the relationship between *DMT1* expression and Hg(II) transport was investigated by RNA-interference in human Caco-2 cells. This also showed that *DMT1* indeed plays an intermediate role in delivering or mediating Hg(II)^20^. However, the Transwell model cannot recapitulate the microenvironment similar to the living intestine, which is critical for forming normal intestinal physiology as well as for the development of intestinal disorders^21,22^.

Organ-on-a-chip models developed from microfluidic devices can replicate the complicated structure and physiological functions of human organs^23–27^. The gut-on-a-chip can cause Caco-2 cells to form a villi-like protrusion by applying mechanical stimulation^28^. The secretory and immune functions of the gut can be partly accomplished by the four differentiated types of cells (absorptive cells, mucous secretory cells, intestinal endocrine cells, and Paneth cells)^29,30^. To support the biological complexity of the gut, sensors were integrated into gut-on-a-chip models to detect indicators or biomarkers in the microenvironment in situ^31–34^. For example, the Ingber group utilized an oxygen-sensitive fluorescent probe for continuously monitoring (for 7 days) oxygen concentration gradient changes within a chip^35^. Monitoring barrier integrity and the inflammatory response (e.g., cytokine secretion) were also realized by fluorescent sensing probes, such as fluorophores, conjugated polymers, deoxyribozymes, and quantum dots^36–39^. However, problems in fluorophore bleaching and service life limit their application in gut-on-a-chip models.

In this study, we developed a gut-on-a-chip integrated with label-free sensors. In addition to reconstructing the human intestinal microenvironment in vitro, sensors in the chip were used for real-time, noninvasive monitoring of the changes in transepithelial electrical resistance (TEER) during cellular spread and in situ measurement of the absorption of Hg(II). Key features of the gut (e.g., the intestinal barrier, villus structures) were confirmed by TEER measurements and immunohistochemical analysis. Hg(II) absorption by intestinal epithelial cells was investigated by 210 nm-thick electrochemical electrodes in the chip using Micro-Electro-Mechanical System (MEMS) technology. The transport mechanism of Hg(II) was explored by the expression of *Piezo1* and *DMT1* under different mechanical stimuli.

## Results and discussion

### Numerical analysis of the gut-on-a-chip

**Fig. 1:**
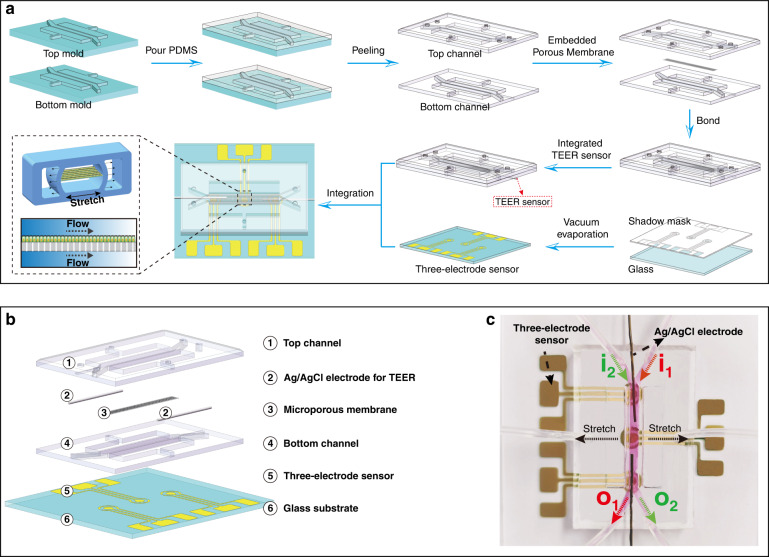
Gut-on-a-chip integrated with sensors. **a** Device fabrication process. **b** Annotated device decomposition diagram. The top and bottom microchannels were separated by the porous membrane. Simultaneous integration of three-electrode sensors and an Ag/AgCl electrode for in situ detection of Hg(II) and TEER. **c** Photograph of the gut-on-a-chip integrated with sensors.

We developed a gut-on-a-chip integrated with TEER sensors and three-electrode electrochemical sensors (Fig. 1). The chip mimics the mechanical behaviors (shear stress and tensile strain) in living intestine by exerting fluid flow and cyclic mechanical stretching.

**Fig. 2:**
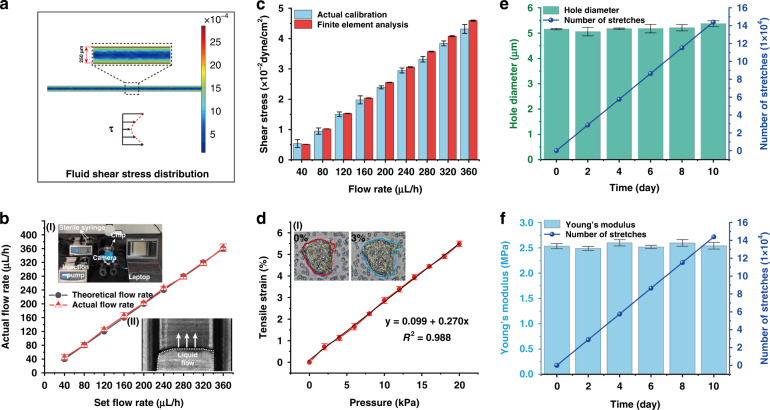
Quantification of physical properties in gut-on-a-chip. **a** FEA results of fluid shear stress in a microchannel. **b** Calibration relationship between the actual flow rate of the culture medium into the gut-on-a-chip and the input flow by the syringe pump. Inset (I): Calibration experimental platform. Inset (II): Microscopic image of fluid flow in the gut-on-a-chip (scale bar, 200 μm). **c** The FEA results of shear stress in the gut-on-a-chip were compared with the actual results (*n* = 3). **d** Quantitation of the mechanical strain produced in the adherent gut epithelial cells as a function of pressure applied by the vacuum controller. Black lines represent linear fitting lines (*y* = 0.099 + 0.270*x*; *R*^2^ = 0.988). Inset (I): Comparison of cell masses on porous membranes before and after stretching by 3% (scale bar, 20 μm). **e** Variation in the pore diameter of the porous film within 10 days under 1% tensile strain. RSD = 1.81% (*n* = 3). **f** The change in Young’s modulus of the porous membrane under 1% tensile strain within 10 days. RSD = 0.07% (*n* = 3).

Shear stress of ~0.02 dyne/cm^2^ produced by fluid flow is crucial for intestinal epithelial morphogenesis^28^. The flow field distribution (Fig. S1) and shear stress field distribution (Fig. 2a, Fig. S2) of the culture medium at different flow rates were obtained by FEA. On the microchannel wall, the shear stress increased from 0.005 dyne/cm^2^ to 0.04 dyne/cm^2^ with increasing flow rate (40–360 μl/h). Furthermore, at a given flow rate, the shear stress parabolically decreases from the wall to the center of the microchannel (Fig. 2a, Fig. S2). Physical measurements of the fluid rate profile were performed to verify the FEA results. We measured the actual flow rate of the perfusion culture medium during operation of the syringe pump (Fig. 2b). The actual shear stress can be obtained based on Newton’s internal friction law (see “Theoretical calculation and simulation of the gut-on-a-chip” in the Methods section). Compared with the FEA results, the maximum relative error was 4.6% (*P* = 0.1) (Fig. 2c). When the flow rate was 160 μL/h, a shear stress of 0.02 dyne/cm^2^ was produced (Fig. 2c).

The distribution of tensile strain and tensile stress in the membrane produced by mechanical stretching can be simulated by FEA (Fig. S3a, b, red-dotted wireframe). Under the same mechanical stretching, the strain and stress distribution along the *X*-axis (white-dotted line) had no significant fluctuation (Fig. S3c, d). Furthermore, the linear relationship between tensile strain and mechanical stretching (which is produced by air pressure) was also obtained from numerical analysis (Fig. S4). Imaging analysis of cell shape (under micromechanical stretching) confirmed that cell deformation increased linearly from 0 to 5.5% when the air pressure increased from 0 to 20 kPa (Fig. 2d), which was in agreement with the above simulation results (Fig. S4). To determine whether the porous membrane will be subjected to fatigue failure under long-term tension, the changes in pore diameter and Young’s modulus were measured under tensile strains (0–5%) for 1.4 × 10^5^ cyclic stretches (10 days). The pore diameter slightly increased from 5.19 ± 0.10 μm to 5.28 ± 0.25 μm, and the Young’s modulus increased from 2.54 ± 0.06 MPa to 2.54 ± 0.05 MPa (Fig. 2e, f and Fig. S5). Therefore, long-term mechanical stretching within a strain range has no significant effect on the physical properties of the pore diameter and Young’s modulus of the porous membrane.

### Effect of the biomimetic gut microenvironment on the intestinal epithelium

**Fig. 3:**
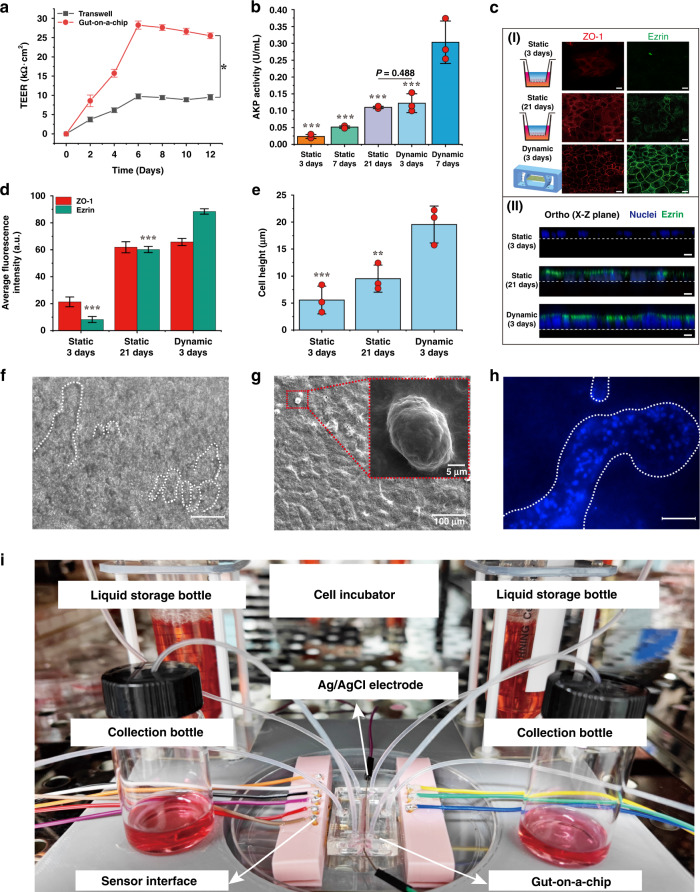
Characteristics of the intestinal epithelium in the gut-on-a-chip. **a** Tight junctional integrity of the epithelium quantified by measuring TEER (*n* = 3). **b** AKP activity under static (3 days, 7 days and 21 days) and dynamic (3 days and 7 days) cultures (*n* = 3; *P < 0.05, **P < 0.01). **c** (I) Confocal fluorescence view of a tight junction protein (ZO-1; red) and brush border protein (ezrin; green) in static (3 days; 21 days) and dynamic cultures (3 days) (scale bar 20 μm). (II) Confocal fluorescence view of vertical cross section of cell monolayer in static (3 days; 21 days) and dynamic culture (3 days) (scale bar 10 μm). **d** Average fluorescence intensity analysis of ZO-1 and ezrin proteins in static and dynamic cultures (3 days) (*n* = 3; ***P < 0.001). **e** Average cell height cultured in static and dynamic cultures (*n* = 3; *P < 0.05). **f** Microscope top-down views of intestinal villi-like structures (scale bar 100 μm). **g** An SEM image of intestinal villi-like structures. **h** A fluorescence microscopic view highlighting the nuclei (DAPI) of intestinal villi-like structures (scale bar 100 μm). **i** A photograph showing the proposed gut-on-a-chip with its monitoring and culturing component.

To explore the effect of mechanical stimulation on cell growth and differentiation, Caco-2 cells were grown either in a Transwell chamber (static culture) or in the gut-on-a-chip (dynamic culture) with flow (160 μL/h; 0.02 dyne/cm^2^) and cyclic mechanical strain (1%; 0.15 Hz) (Supplementary video 2). Transepithelial electrical resistance (TEER) is a widely accepted quantitative technique to evaluate the integrity of tight junctions during the culture of endothelial and epithelial monolayers^40^. The TEER of the cells grown in the Transwell increased during the first 6 days and was saturated for the subsequent 6 days. The peak TEER value of the cells in the gut-on-a-chip was 28.2 kΩ cm^2^, which was threefold higher than that of the cells in the Transwell culture (Fig. 3a). To determine whether fluid flow and mechanical strain alter cytodifferentiation, we analyzed the catalytic activity of alkaline phosphatase (AKP) in Caco-2 cells. AKP activity increased >2-fold in Caco-2 cells cultured for 21 days compared to cells cultured for 7 days in static culture. Importantly, cells under fluid flow and mechanical strain showed greatly accelerated AKP activity, producing a nearly sixfold increase in AKP activity after only 7 days in culture (Fig. 3b). In addition, there was no significant difference in the secretion of AKP between dynamic (3 days) and static (21 days) conditions (*P* = 0.488).

We further analyzed the expression of the tight junction protein ZO-1 and brush border protein ezrin in static culture (3 days; 21 days) and dynamic culture (3 days). Caco-2 cells displayed tight junctions and brush borders, as shown by immunofluorescence staining of ZO-1 (red staining in Fig. 3c(I)) and ezrin (green staining in Fig. 3c(I)). Compared with static conditions for 3 days, cells in static culture for 21 days or dynamic culture for 3 days formed more confluent polygonal epithelial monolayers with well-developed tight junctions. However, ezrin in biomimetic dynamic culture (3 days) was 1.6-fold higher than that in static culture (21 days) (Fig. 3d). The confocal cross-sectional imaging (Fig. 3c(II)) of cells showed that the height of dynamically cultured (3 days) cells was 2- to 3-fold times higher than that of statically cultured (21 days) cells (19.7 ± 3.3 μm vs. 9.6 ± 2.5 μm) (Fig. 3e). Interestingly, we found that the originally planar columnar epithelium spontaneously grew to form undulations and folds in dynamic culture (Fig. 3f, g). This folded structure was composed of many cells. Cells showed a tubular-like distribution (Fig. 3h). These folds were similar in shape to intestinal villi. The gut-on-a-chip and its monitoring and culturing are shown in Fig. 3i and Fig. S6.

The above results revealed that the growth and differentiation of the cell monolayer could be accelerated under reasonable mechanical stimulation optimized by numerical analysis. The cells grown on the established gut-on-a-chip system exhibited intestinal villus-like structures and maintained the integrity of the tissue barrier, representing the key features of the human intestine in a physiologically relevant manner.

### Damage to intestinal epithelial cells induced by Hg(II)

**Fig. 4:**
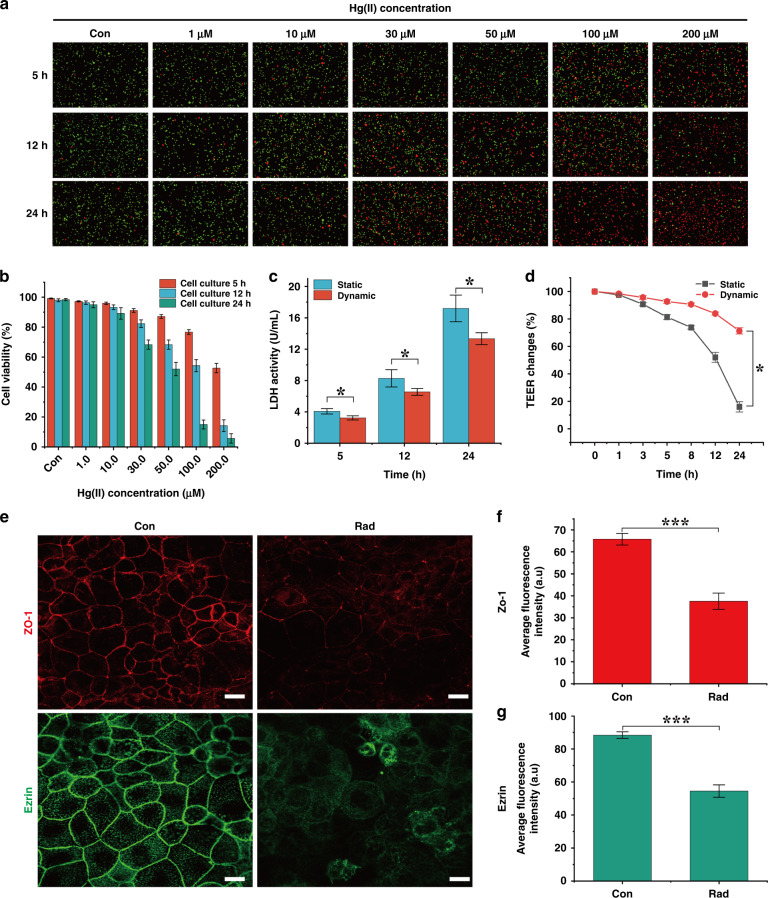
Damage to cells caused by Hg(II). **a** Immunofluorescence of cells exposed to different concentrations of Hg(II) for 5, 12, and 24 h in static culture (red: dead cells; green: living cells). **b** Changes in cell activity under the treatment of different concentrations of Hg(II). The cell activity remained above 80% within 24 h under low concentrations of Hg(II) (1, 10 μM) (*n* = 3). **c** Epithelial cells were exposed to 100 μM Hg(II), and LDH was detected at 5, 12‘ and 24 h. Within 24 h, the results of LDH detection showed that the expression of LDH in static samples was 1.3-fold higher than that in dynamic samples (*n* = 3; *P < 0.05). **d** The change rate of TEER in static and dynamic cultures of cells exposed to 100 μM Hg(II) within 24 h. The TEER under dynamic conditions was 4.4-fold higher than that under static conditions (*n* = 3; *P < 0.05). **e** In the absence or presence of Hg(II) (100 μM), confocal immunofluorescence of ZO-1 (red) and ezrin (green) protein was observed (scale bar 20 μm). **f**, **g** Analysis of the average fluorescence intensity of ZO-1 and ezrin proteins after epithelial cell injury. Compared with the control group, the fluorescence intensity of ZO-1 and ezrin decreased by 1.8-fold (*n* = 3; ***P < 0.001).

To evaluate the damaging effect of Hg(II) on Caco-2 cells, we first exposed cells in static culture to different concentrations of Hg(II). With increasing Hg(II) concentration and culture time, the amount of cell death increased (Fig. 4a). The cell activity remained above 80% within 24 h under low concentrations of Hg(II) (1, 10 μM). There was no significant difference (*P* > 0.05) in cell viability compared to the control group (without Hg(II) treatment), which ensured the integrity of the cell barrier (Fig. 4b). In contrast, high concentrations (100 μM) of Hg(II) damaged the tight junctions between cells, causing exfoliation and cell death (Fig. S7). To compare the damage of Hg(II) on the cell monolayer in static and dynamic cultures, we detected the changes in LDH activity and TEER within 24 h after adding Hg(II) (100 μM). In both culture conditions, the expression of LDH increased with time. This showed that Hg(II) caused damage to cellular membranes in both cultures. However, the degree of injury was different. The expression of LDH under static conditions was 1.3-fold higher than that under dynamic conditions after treatment with Hg(II) for 5, 12, and 24 h (Fig. 4c). The detection of TEER also showed the same result: both culture conditions showed a downward trend in TEER, but the TEER in dynamic culture was always higher than that in static culture (Fig. 4d). This could be because the villus-like structure contributes to the formation of a stronger barrier integrity in the cell monolayer under mechanical stimulation. Meanwhile, mucus secretion at the top of the villi was enhanced to increase self-protection ability^29^ and reduce the damage caused by Hg(II) to the cells. Because ZO-1 and ezrin are the key proteins that make up the cell barrier and intestinal villus structure, respectively, we further analyzed the damage caused by Hg(II) to these two proteins in dynamic culture. Compared with the control group, the staining of the two proteins did not show a clear boundary, and the distribution was uneven (Fig. 4e). In the damaged epithelial cells, the expression of ZO-1 and ezrin decreased by 1.8-fold (Fig. 4f, g). The decrease in ZO-1 protein expression was the main reason for the decrease in TEER. We concluded that the effect of Hg(II) on dynamic culture was less than that on static culture, and a low concentration of Hg(II) (≤10 μM) had no significant effect on cell survival. In addition, Hg(II) could destroy the tight junction proteins between cells, resulting in the disappearance of cell barrier function.

### In situ detection of the absorption of Hg(II) in the gut-on-a-chip

**Fig. 5:**
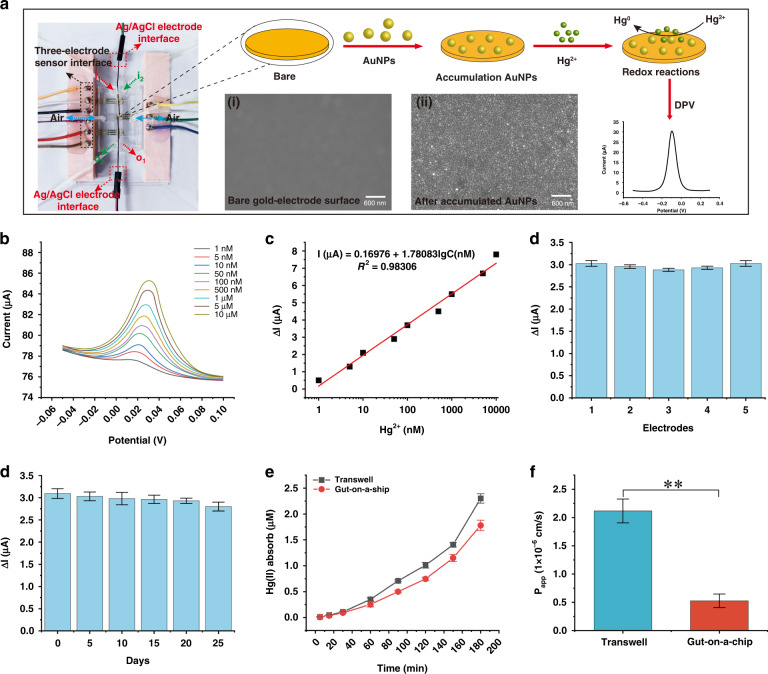
Absorption of Hg(II) by epithelial cells. **a** Photograph of the sensor-integrated gut-on-a-chip and schematic of the AuNP modification process. Insets (i and ii): SEM images of the gold electrode surface with and without AuNPs. **b** DPV responses of the three-electrode sensor with different concentrations of Hg(II) from 1 nM to 10 µM. **c** The fitting result between ∆I (the difference between the peak current and background current) and the concentration of Hg(II); ΔI = 0.16976 + 1.78083 lg C (*R*^2^ = 0.983). **d** Reproducibility verification. Five sensors were randomly selected to detect the DPV response of 50 nM Hg(II), the relative standard deviation was 4%, and the electrode had good consistency (*n* = 3). **e** Stability of the sensor during 25-day storage. The peak current decreased slightly with increasing time, and the peak current on the 25th day was 86% of the initial value; thus, the electrode has good stability (*n* = 3). **f** Hg(II) absorption curve of the Transwell and gut-on-a-chip during 180 min of culture. During the 180-minute detection, the concentrations of Hg(II) absorbed by intestinal epithelial cells in Transwells and gut-on-a-chip were 2.3 and 1.8 μM, and the absorption rates were 22% and 17.8%, respectively (the initial concentration of Hg(II) in the top chamber/channel was 10 μM) (*n* = 3). **g** Comparison of the P_app_ value of Hg(II) absorbed by the Transwell and gut-on-a-chip systems. The P_app_ value of the Transwell was fourfold higher than that of the gut-on-a-chip. (*n* = 3; **P < 0.01).

To realize in situ detection of Hg(II) absorption by intestinal epithelial cells, an electrochemical sensor array was integrated into the gut-on-a-chip (Fig. 5a). The schematic in Fig. 5a shows the modification steps of gold nanoparticles (AuNPs) for detecting Hg(II) based on the principle of the redox reaction. The SEM images in Fig. 5a show that AuNPs (diameter range of 40–50 nm (Fig. S9b)) were successfully modified on the surface of the electrode.

To optimize the differential pulse voltammetry (DPV) response for Hg(II) of the sensor, AuNPs deposited for different times (0, 75, 100, 125, 150, 175, and 200 s) were investigated. When the deposition time increased from 0 to 175 s, the peak current showed an increasing trend because AuNPs enhanced the conductivity and the specific surface area of the electrodes. Continuing to increase the deposition time, the current had no obvious increase because of the saturation of the active sites on the surface of the sensor (Fig. S9c, d). Hence, 175 s was selected as the deposition time for subsequent testing.

Calibration for detecting Hg(II) of the sensor was investigated. The peak current of Hg(II) occurred at a potential of 20 mV, and it continuously increased from 0.5 to 7.8 μA for Hg(II) concentrations from 1 nM to 10 μM (Fig. 5b). The fitting curve of Hg(II) was ΔI = 0.16976 + 1.78083 lg C (R^2^ = 0.983), where ∆I is the difference in the oxidation peak current and lg C represents the log concentration of Hg(II) (Fig. 5c). The calculated sensitivity for Hg(II) was 1.78 μA/nM, and the detection limit was 0.1 nM. The reproducibility of the sensor was evaluated by repetitive measurement of 50 nM Hg(II). For five different sensors prepared in the same batch, the RSD was 2.1% for Hg(II) (Fig. 5d, S9a). The stability of the proposed sensor was studied by detecting the DPV responses of the electrode to 50 nM Hg(II). The DPV response was reduced by 16% after storage for 25 days (Fig. 5e, S9b).

The sensor array in the chip was used to continuously monitor the Hg(II) absorption of the Caco-2 cells cultured in situ in the gut-on-a-chip. Culture medium mixed with 10 μM Hg(II) was perfused into the chip. Hg(II) absorbed by cells showed an upward trend and reached 1.8 μM (a rate of 17.8%) after 180 min of treatment, whereas Hg(II) absorption in the Transwell was 2.3 μM (a rate of 22%) (Fig. 5f, S10). The transport permeability (P_app_) to Hg(II) of the Transwell was fourfold higher than that of the gut-on-a-chip (Fig. 5g).

In vivo testing concluded that Hg(II) absorption is <15.0%^17^, which was lower than that deduced from Caco-2 cells (17.8%). It should be considered that in vivo studies are characterized by the presence of luminal factors (bile salts, food components, etc.) that do not exist in our chip, which could affect Hg(II) transport across the epithelial cells of the intestine^16^.

### Absorption of Hg(II) under different mechanical stimuli

**Fig. 6:**
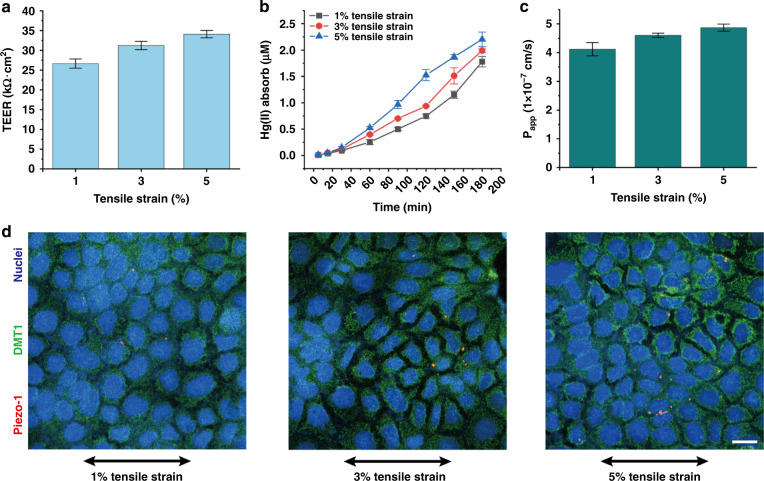
Exploring the transport mechanism of Hg(II). **a** The TEER value of the cell monolayer on the 7th day under different mechanical stretching conditions (1%, 3%, 5%). The TEER values were 26.65 ± 1.17 kΩ cm^2^, 32.21 ± 1.05 kΩ cm^2^ and 34.10 ± 0.93 kΩ cm^2^, and the cell barrier ability was increased by 20.86% and 27.95% (1% mechanical stimulation as the control group) (*n* = 3). **b** Changes in intestinal epithelial cell monolayer absorption of Hg(II) within three hours under different mechanical stretching conditions (1%, 3%, 5%). The concentrations of Hg(II) absorbed by cells under three mechanical stretching stimuli were 1.78 ± 0.11 μM, 1.98 ± 0.03 μM, and 2.20 ± 0.14 μM (*n* = 3). **c** The change in the P_app_ value under different mechanical stretching (1%, 3%, 5%). Compared with 1% mechanical stimulation, the ability of cells to absorb Hg(II) increased by 11.65% and 17.96% under 3% and 5% mechanical stimulation, respectively (*n* = 3). **d** The expression of *Piezo1* protein and *DMT1* protein under different mechanical stretching conditions (*Piezo1*: red; *DMT1*: green; nucleus: blue) (scale bar 20 μm).

Finally, to explore the transport mechanism of Hg(II), we assessed the Hg(II) absorption and the expression of key proteins (*Piezo1* and *DMT1*) under different tensile strains (1%, 3%, 5%). We first studied the effects of different tensile strains on the cell barrier (TEER value change). For tensile strains of 1%, 3%, and 5%, TEER values were 26.65 ± 1.17 kΩ cm^2^, 32.21 ± 1.05 kΩ cm^2^ and 34.10 ± 0.93 kΩ cm^2^, respectively (Fig. 6a). Values greater than 25 kΩ cm^2^ indicate the formation of the complete cell barrier. The Hg(II) concentrations absorbed by the cells were 1.78 ± 0.11 μM, 1.98 ± 0.03 μM, and 2.20 ± 0.14 μM for tensile strains of 1%, 3%, and 5%, respectively (Fig. 6b). Compared with 1% tensile strain, Hg(II) absorption increased by 11.23% and 23.59% under tensile strains of 3% and 5%, respectively, and P_app_ increased by 11.65% and 17.96% under 3% and 5% tensile strain, respectively (Fig. 6c). Therefore, an increase in mechanical stimulation in a certain range promotes Hg(II) absorption by intestinal cells. For further verification, immunohistochemical analysis showed that the expression of *Piezo1* and *DMT1* was upregulated with increasing mechanical stimulation (Fig. 6d). Compared to 1% tensile strain, for tensile strains of 3% and 5%, *Piezo1* expression increased by 21.7% and 91.35%, respectively, and *DMT1* protein expression increased by 3.9% and 9.4%, respectively (Fig. S11). These results showed that when mechanical stimulation increased, the absorption of Hg(II) by intestinal epithelial cells increased, and *Piezo1* and *DMT1* also showed a positive correlation with mechanical stimulation.

## Conclusions

In this study, we developed a gut-on-a-chip integrated with TEER sensors and multiple electrochemical sensors for simulating Hg(II) transport in the human intestine in vitro. This chip can dynamically observe the formation of the physical intestinal barrier and monitor the transport of Hg(II) in real time. Microscopic imaging, immunohistochemical analysis, and real-time detection with sensors showed that the Caco-2 cell monolayer in the gut-on-a-chip (160 μL/h; 1%) was highly differentiated, forming a complete cellular barrier. It was also demonstrated that Hg(II) could damage the tight junctions between cells, causing cells to undergo exfoliation and die. Meanwhile, the absorption level of Hg(II) in the gut-on-a-chip was close to the intestinal absorption level in vivo (17.8% vs. 15.0%). In addition, when mechanical stimulation increased to 3% and 5%, Hg(II) absorption increased by 11.23% and 23.59%, respectively, and *Piezo1* and *DMT1* also showed a positive correlation. This methodology can also be used to understand the mechanism of human intestinal diseases and promote the development of personalized drugs.

## Methods

### Fabrication of the gut-on-a-chip integrated with sensors

#### Fabrication of a gut-on-a-chip

The gut-on-a-chip was fabricated by polydimethylsiloxane (PDMS; 184 Silicone Elastomer, Dow Corning Co., USA). The chip consists of three parts: the top and bottom parts with microchannels (2.0 mm width × 0.25 mm height) and the middle porous membrane (20 μm thick; 5 μm diameter of the pore; E.motion membrane; Sweden). The porous membrane separates the top and bottom parts, playing a role in constructing the tissue interface. First, the base elastomer and curing agent of PDMS were mixed with a weight ratio of 10:1 (wt/wt). After degassing for 30 min, PDMS was poured into the mold with a microchannel pattern. The top and bottom parts with microchannels were made after curing at 70 °C for 4 h. To deliver the cell culture medium, openings were punched in the top part. Then, the top part, porous membrane, and bottom part were bonded together by oxygen plasma for 120 s (CY-P2L-B, CY Scientific Instrument CO., Ltd, China). Finally, a stainless steel thin tube (internal diameter: 0.8 mm; external diameter: 1.0 mm) was used to connect the microchannels in the chip with the silica gel capillary (internal diameter: 1.0 mm; external diameter: 1.5 mm). Flow rates and mechanical stretch were controlled using a high-precision syringe pump (LSP02-1B, Baoding Ditron Electronic Technology CO., Ltd).

#### Integration of TEER and electrochemical sensors into the gut-on-a-chip

##### Integration of TEER sensor

The TEER sensors consist of two Ag/Cl electrodes (diameter 0.2 mm), which were used to detect the integrity of the cell barrier. After the preparation of the gut-on-a-chip, the electrode was embedded into the top and bottom sides of the porous membrane.

##### Integration of electrochemical sensors

The electrochemical sensor was a three-electrode system sensor fabricated by MEMS technology. The sensor was composed of a glass substrate, Cr (10 nm) and Au (200 nm) (Fig. S8a).

The bottom part of the gut-on-a-chip was cut along the edge of the microchannel by using a surgical blade. Glass slides with three-electrode sensors and the gut-on-a-chip were bonded and sealed by oxygen plasma for 120 s. For details of the electrochemical sensor fabrication, please see Section 1.1 of the Supporting Information.

### Theoretical calculation and simulation of the gut-on-a-chip

COMSOL Multiphysics 5.5 (trial version) was used to simulate the mechanical tensile stress and fluid shear stress (FSS) in the microchannel. The geometric size of the model was the same as the actual size of the chip.

#### Calculation and simulation of tensile stress of the porous membrane

To simulate the stress‒strain relationship during the stretching process of the porous membrane in the chip, the equilibrium differential equation (Eqs. (1) and (2)) was used to solve the stress‒strain relationship in the Structural Mechanics Module in COMSOL:

1 $$\rho \frac{{\partial ^2\overrightarrow u }}{{\partial t^2}} + d_a\frac{{\partial \overrightarrow u }}{{\partial t}} - \nabla \cdot \widetilde \sigma = \overrightarrow f$$

2 $$\nabla = \frac{\partial }{{\partial x}}\overrightarrow t + \frac{\partial }{{\partial x}}\overrightarrow j$$

where $\rho$ is the density, *d*_*a*_ is the damping coefficient, $u$ is the displacement, *t* is the time, $\sigma$ is the stress, ∇ is the differential operator symbol and $f$ is the volume force.

For the steady-state simulation in this study, the above equilibrium differential equation can be simplified as Eq. (3):

3 $$- \nabla \cdot \widetilde \sigma = \overrightarrow f$$

#### Calculation and simulation of fluid shear stress in the microchannel

The perfusion rate of the culture medium was determined to provide the appropriate fluid shear stress (0.02 dyne/cm^2^). The flow field was obtained by solving the steady-state incompressible Navier‒Stokes equation. The microchannel walls were set as a no-slip boundary condition. The FSS distribution along the microchannel wall can be computed using Eq. (4):

4 $$\tau = \frac{{{{{\mathrm{d}}}}{\it{U}}}}{{{{{\mathrm{d}}}}{\it{z}}}}\mu$$

where $\tau$ is the shear stress (dyne/cm^2^), $\mu$ is the dynamic viscosity (g/cm·s), and $\frac{{{{{\mathrm{d}}}}{\it{U}}}}{{{{{\mathrm{d}}}}{\it{z}}}}$ is the shear rate (obtained by solving the Navier‒Stokes equation in COMSOL).

The microchannel is a rectangular structure. To verify the simulation result, Eq. (5) was obtained based on Newton’s internal friction law of fluid mechanics.

5 $$\tau = \frac{{6\mu Q}}{{Wh^2}}$$

Here, $Q$ is the volumetric flow rate (cm^3^/s), $W$ is the channel width (cm), and $h$ is the channel height (cm).

### Young’s modulus measurement

To determine whether the porous membrane undergoes fatigue failure under long-term tension, we measured the changes in porous diameter and Young’s modulus under different tensile strains (0 – 5%) for 1.4 × 10^5^ cycles (10 days).

The mechanical strain (1%; 0.15 Hz) of the porous membrane was applied continuously for 10 days. The tensile test of the porous membrane was carried out every day by using an electronic universal testing machine (AGS-X5KN, SHIMADZU, Japan), and the change in Young’s modulus was measured.

Different mechanical tensile strains (0–5%) were applied to the porous membrane and after 10 days of continuous application. The change in the Young’s modulus of the porous membrane was measured by using an electronic universal testing machine.

### Cell culture

The human colon adenocarcinoma cell line Caco-2 (RuYao Biotechnology, China) was cultured in T25 flasks in DMEM (Gibco, Waltham, MA, USA), 10% fetal bovine serum (FBS; A3160801, Gibco, USA), and 1% penicillin/streptomycin (MA0110, Meilunbio, China). Caco-2 cells between passages 5 and 10 were used for all experiments. Cells were routinely tested for mycoplasma contamination and found to be negative. For details of the cell culture methods, please see Section 1.4 of the Supporting Information.

### Transepithelial electrical resistance measurements

To detect the change in cell monolayer transepithelial electrical resistance, the Ag/AgCl electrode integrated in the gut-on-a-chip was connected to an electrochemical workstation (PGSTAT302N, Herisau, Switzerland). The change in resistance was recorded by electrochemical impedance spectroscopy (EIS). The TEER value was calculated using the following formula:

6 $${\rm{TEER}} = \left( {R_1 - R_0} \right) \cdot S$$

where $R_1$ is the actual measured value, $R_0$ is the baseline resistance value measured in the absence of cells, and *S* is the surface area of the cell culture.

**Fig. 7:**
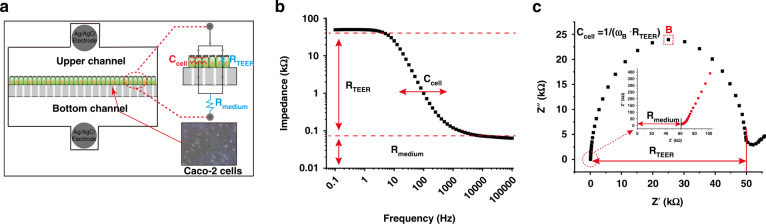
TEER detection principle. **a** Schematic diagram and equivalent circuit diagram for the detection of TEER in intestinal epithelial cells by EIS. **b**, **c** Bode curve and Nyquist curve for TEER detection by EIS. Through these two curves, the distribution of components in the equivalent circuit diagram can be shown. In the Nyquist curve, the cell capacitance can be calculated by using the angular frequency ω_B_ of the semicircle vertex **B**. The formula is marked in (**c**).

EIS across a wide frequency spectrum provides a more accurate TEER value and capacitance of the cell layer than the traditional DC or single-frequency AC measurement method. Figure 7a–c shows a typical equivalent circuit model that was used to analyze the impedance spectrum of the cells grown in static and dynamic cultures. This model accounts for electrical current can flow through the junctions among cells (paracellular route) and through the cell membrane (transcellular route). The tight junction protein ZO-1 in the paracellular route contributes to ohmic resistance (R_TEER_) in the equivalent circuit. Each lipid bilayer in the transcellular route contributes to the electrical capacitance (C_cell_) in the circuit. The resistance of the cell culture medium (R_medium_) was also considered. The value of each component could be calculated from the Bode curve (Fig. 7b) and the Nyquist curve (Fig. 7c). For the explanation and other details of the principle of electrochemical impedance spectroscopy (EIS) for the detection of TEER, please see Section 1.5 of the Supporting Information.

### Evaluation of alkaline phosphatase activity

Alkaline phosphatase (AKP) is a specific protein secreted by Caco-2 cells in the formation of brush marginal microvilli. AKP is commonly and quantitatively used to evaluate human intestinal epithelial cell functionality. The activity of AKP was measured by an alkaline phosphatase (AKP) kit (BC2145, Beijing Solarbio Science & Technology Co., Ltd, China).

For the cells in the Transwell, fresh complete medium was added to cells that had been cultured for 7 and 21 days, incubated for 4 h (37 °C; 5% CO_2_), and transferred into 96-well plates. The absorbance was recorded by a microplate reader with a wavelength of 510 nm (Multiskan FC; Thermo Scientific, USA).

For cells in the gut-on-a-chip, the epithelial cells were continuously cultured for 7 days under mechanical stimulation. To detect the AKP activity of the gut-on-a-chip cells, fresh complete medium continuously flowed through the microchannel at a flow rate of 50 μL/h for 4 h. Metabolic fluid was collected at the outlet of the chip and doubly diluted. The measurement procedure was performed as described above.

### The viability of Caco-2 cells exposed to Hg(II)

To investigate the viability of Caco-2 cells exposed to different Hg(II) concentrations, a live/dead cell staining kit (MA0361, Dalian Meilun Biotechnology Co., Ltd, China) was used for imaging. A stock of 1 mM Hg (II) was diluted to 0.5, 1, 10, 30, 50, 100, and 200 μM in serum-free culture medium, and the effect of each concentration was tested for 5, 12, and 24 h. A 200 μL live/dead cell staining kit was incubated for 3 min, followed by treating Caco-2 cells with trypsin/EDTA (0.25%, 25200-056, USA). An inverted fluorescence microscope (Nikon Eclipse Ti2) was used to record live cells (green fluorescence) and dead cells (red fluorescence) at wavelengths of 495 and 652 nm, respectively. ImageJ (National Institutes of Health) software was used to count live/dead cells and calculate cell viability.

### Measurement of lactate dehydrogenase activity

To evaluate the cell damage caused by Hg(II), the activity of lactate dehydrogenase (LDH) was quantified. LDH is an important enzyme of the anaerobic metabolic pathway and is a widely distributed enzyme in cells. LDH leaks into the cell culture medium when cellular membranes become permeable. The activity of LDH was measured by a lactate dehydrogenase kit (A020-2, NJJCBIO, China). Serum-free medium containing 100 μM Hg (II) was added to the top chamber of the Transwell and the upper channel of the gut-on-a-chip. The cell culture medium at 5, 12 and 24 h was placed in a centrifuge tube and then centrifuged for four minutes (1200 r/min). The supernatant was removed, and the enzyme activity was detected according to the instructions of the LDH kit (A020-2, NJJCBIO, China). The absorbance of LDH at 450 nm was recorded using a multimode plate reader (Multiskan FC; Thermo Scientific). The LDH activity was evaluated in triplicate for each group and calculated after subtracting the background absorbance of the 96-well plate.

### Effect of different solutions on the detection of Hg(II)

To verify the effect of different solutions on the detection of mercury ions, combined with the needs of cell growth, D-Hanks buffer (MA0039; Meilunbio, China), serum-free medium (DMEM + 1% penicillin/streptomycin) and complete medium (DMEM + 1% penicillin/streptomycin + 10% FBS) were selected for verification. The peak current of Hg(II) in three kinds of solutions was detected by differential pulse voltammetry (DPV) in an electrochemical workstation (Fig. S12).

### The calibration curves of Hg(II)

To obtain the calibration curve of the sensor for detecting Hg(II), nine concentrations (1 nM, 5 nM, 10 nM, 50 nM, 100 nM, 500 nM, 1 μM, 5 μM, 10 μM) of Hg(II) solution were detected by DPV. The DPV measurements were performed with a pulse period of 0.5 s, a pulse width of 0.05 s, and a pulse amplitude of 25 mV. The potential range was −0.05–0.1 V. Each concentration was measured at least three times, and the peak current was recorded. The linear regression equation between current and concentration was obtained by Origin 2020 software.

### Verification of microelectrode consistency and stability

Consistency and stability are important characteristics of sensors. To detect the consistency of the microelectrodes, five microelectrodes were used to detect the DPV response to 50 nM Hg(II). To verify the stability of the electrode, the response of DPV to 50 nM Hg (II) was measured every five days for a total of 25 days.

### Absorption detection of Hg(II) in transwells and gut-on-a-chip

Epithelial cells in the gut-on-a-chip form a complete barrier after culturing for 7 days. A serum-free culture medium containing 10 μM Hg(II) is perfused (160 μL/h) through a syringe pump to the top channel of the gut-on-a-chip. The perfusion was stopped after the top channel was filled with culture medium. The bottom channel was perfused with D-hanks buffer without Hg(II) in the same way. An electrochemical workstation was used to connect the three-electrode sensor, and the mercury ion content in the bottom channel was detected every 30 min.

In the Transwell, 500 μL serum-free culture medium containing 10 μM Hg(II) was added to the insertion chamber, 1000 μL of D-hanks buffer was added to the bottom chamber, and the bottom solution was removed every 30 min for mercury ion detection.

### Detection by ICP‒MS

To verify the accuracy of the electrochemical sensor in detecting Hg(II), we collected metabolic fluid at different time points (60, 120, and 180 min) in the gut-on-a-chip. An electrochemical sensor and an inductively coupled plasma mass spectrometer (ICP‒MS, NexION 1000, USA) were used to detect Hg(II) in metabolic solution. The comparison of the test results is shown in Fig. S13.

### Transport calculation of apparent permeability

The apparent permeability coefficients (Papp; cm/s) were calculated from Eq. (7):

7 $$P_{app} = \left( {dC/dt} \right)\left( {V_r/AC_0} \right)$$

where $dC/dt$ is the flow (mg/mL/s) determined from the linear slope of the equation defining the variation in mercury concentration (corrected for dilution) versus time; $V_r$ is the volume of the receptor compartment (mL); *A* is the surface of the cell monolayer (cm^2^); and $C_0$ is the initial mercury concentration (mg/mL).

### Morphological analyses

Morphological analyses were performed using at least three independent gut-on-a-chip samples at each time interval. The villus microarchitecture was studied using laser scanning confocal microscopy (LSCM) (Zeiss LSM880). The DIC module in LSCM was used to analyze the cell surface morphology, and the immunofluorescence module was used to image the ZO-1 protein (Ex: 652 nm, Em: 668 nm) and ezrin protein (Ex: 495 nm, Em: 519 nm). The acquired images were analyzed using ZEN 3.3 and ImageJ software.

### Immunofluorescence microscopy

The Caco-2 cell monolayer was fixed with 3.7% formaldehyde (MA0192, Meilunbio, China) in phosphate-buffered saline (PBS; PWL050, Meilunbio, China) for 15 min, washed twice for 5 min with PBS and permeabilized with 0.3% Triton X-100 (Cat# T8002, Solarbio, China) in PBS for 10 min. After washing with 4% FCS in PBS, the cells were incubated with blocking solution (2% FCS, 2% bovine serum albumin (BSA) (A8010, Solarbio), 0,1% Tween 20 (Sigma# P9416) in PBS) for 45 min. Subsequently, the cells were incubated with primary antibodies for 60 min or at 4 °C overnight, washed three times, incubated with secondary antibodies for 30 min and washed three times with 4% FCS in PBS. For more information about the antibodies and details used in the immunofluorescence experiment, please see Section 1.6 of the Supporting Information.

### Statistical analyses

All experiments were carried out at *n* = 3–6, and the results are presented as the mean ± standard error of the mean (s.e.m.). Data analysis was performed with one-way analysis of variance with Tukey’s HSD post hoc tests using GraphPad Prism 9 (trial version) and Origin 2021 (student version) software. Statistical analysis between two conditions was performed by an unpaired Student’s *t* test. *P* values of <0.05 were considered to be statistically significant (*P < 0.05, **P < 0.01, ***P < 0.001).

## Supplementary information

Supporting Information

Supplementary video.1

Supplementary video.2
