# Supplementary material for: Gut-on-a-chip for exploring the transport mechanism of Hg(II)
Source: Microsyst Nanoeng. 2023 Jan 1;9:2. doi: 10.1038/s41378-022-00447-2 (PMC9805456; doi:10.1038/s41378-022-00447-2)
Supplement: Supplementary file 1 — Supporting Information [file 41378_2022_447_MOESM1_ESM.docx]

# Supporting Information

Gut-on-a-chip for Exploring Transport Mechanism of Hg (Ⅱ)

**Li Wang^1,2*^, Junlei Han^1,2^, Weiguang Su^1,2^, Anqing Li^1,2^, Wenxian Zhang^1,2^, Huimin Li^1,2^, Huili Hu^3,4^, Wei Song^5^, Chonghai Xu^1,2^, Jun Chen^1,2^**

**^1^**School of Mechanical Engineering, Qilu University of Technology (Shandong Academy of Sciences), Jinan 250353, China

**^2^**Shandong Institute of Mechanical Design and Research, Jinan 250353, China

**^3^**The Key Laboratory of Experimental Teratology, Ministry of Education and Department of Genetics, School of Basic Medical Sciences, Shandong University, 250012, Jinan, China

**^4^**The Research Center of Stem Cell and Regenerative Medicine, School of Basic Medical Sciences, Cheeloo Medical College, Shandong University, 250012 Jinan, China

**^5^**Department of Oncology, Shandong Provincial Hospital Affiliated to Shandong University, Jinan, Shandong 250021, China

* Corresponding author: [liwang@qlu.edu.cn](mailto:liwang@qlu.edu.cn)

**Highlights:**

- A gut-on-a-chip integrated with sensors was developed for exploring the transport mechanism of Hg(Ⅱ) in the intestine.
- The gut-on-a-chip can in situ and simultaneously monitor the intestinal barrier function and Hg(Ⅱ) absorption.
- YAP signal pathway may dominate Hg(Ⅱ) transport and absorption due to the positive correlations among the mechanical stimulation, the expression of Piezo-1 and *DMT1*.

**Keywords：** Gut-on-a-chip, Integrated sensors, Mercury ions, Transport mechanism

# 1. Experimental and Methods

## 1.1 Fabrication of microelectrode

The designed microelectrode for detection of Hg(Ⅱ) was three-electrodes configuration: Au reference electrode (RE), Au counter electrode (CE), and Au working electrode (WE). The glass substrate is cleaned by ultrasonic cleaning with anhydrous ethanol for 30 minutes, and then the surface is blown dry with nitrogen. Then the glass surface was cleaned by oxygen plasma, and the microelectrode was prepared by shadow mask technology. In this process, metal layers were deposited over a shadow mask that has apertures in the metal film of 0.30 mm thickness. Cr (10 nm)/Au (200 nm) film stacks were prepared by vacuum evaporation on a clean glass substrate with a shadow mask attached to them. Cr layer acts as the adhesion promoter for the gold film. The gold electrode was washed with ethanol and deionized water, then soaked in piranha solution (H2O2(v)/H2SO4(v) = 1/3) for 5 minutes to clean the surface of the electrode, then washed with deionized water and dried with nitrogen.

## 1.2 Quantification of tensile strain

For the determination of the vacuum cycle pressure, we first set the properties of the porous membrane material through the finite element simulation analysis, and apply the force on both sides of the porous membrane to determine the force needed to produce the corresponding deformation. In the actual experiment, we realize the cyclic stretching of the porous membrane by using the injection pump to push and pull the 1ml aseptic syringe. To verify the accuracy of the simulation results, we connect the gas pipeline to the barometer and the gut-on-a-chip at the same time. The intestinal organ chip was placed under the microscope to determine multiple marker points. Push and pull the syringe 0.1ml each time, and continue to increase, measure the deformation of the marked points on the porous membrane, and determine the average strain of the porous membrane.

## 1.3 Quantification of perfusion flow of culture medium

First of all, according to the size of the design, the finite element analysis is carried out to determine the theoretical flow rate of the culture medium. At the same time, to determine the accuracy of the flow rate of the injection pump in the process of low-speed injection, we set different flow rates and injected continuously for 12 hours to collect the culture medium discharged by the chip and measure the volume to determine the actual flow in the intestinal chip. Using a high-precision injection pump and a 2 mL aseptic syringe, the culture fluid was slowly infused into the upper and lower layers of the gut-on-a-chip to simulate the fluid flow and shear stress in the intestine. First, the aseptic syringe was sucked into the complete culture medium of 2 mL and fixed on the injection pump. A sterile capillary is then used to connect the syringe to the upper and lower channels of the gut-on-a-chip. Start the injection pump and fill the whole channel with culture fluid to eliminate the influence of internal factors (such as bubbles) on the experimental results. By setting the parameters of the injection pump, the accuracy of the perfusion culture medium of the injection pump was calibrated in the flow range of 10 μL/h-400 μL/h, and the perfusion time was 12 h. Finally, the actual perfusion flow value of the culture medium was calculated by calculating the volume of the effluent.

## 1.4 Cell culture

### 1.4.1 Gut-on-a-chip culture

Human Caco-2 intestinal epithelial cells were purchased from RuYao Biotechnology(Zhejiang; China). Caco-2 cells were cultured in high-glucose Dulbecco’s modified Eagle’s medium (DMEM, Gibco, Waltham, MA, USA) supplemented with 10% fetal bovine serum (FBS, Gibco). All cells were cultured at 37 ℃ in a humidified atmosphere of 5% CO_2_. After microdevice fabrication and assembly, the tubing and microfluidic channels were sterilized by flowing 75% (v/v) ethanol through the device and drying the entire system in a 60 ℃ oven. The dried devices were then exposed to ultraviolet light and ozone simultaneously for 30 min. An ECM solution containing rat type I collagen (5 mg/ml, Solarbio, China) and Matrigel (8 mg/ml, Corning, USA) in serum-free DMEM was injected into the microchannels and incubated at 37 ℃ for 2h, after which the microchannels were perfused with culture medium. Caco-2 cells harvested with trypsin/EDTA solution (0.05%; Gibco) were plated on the top surface of the ECM-coated porous membrane (3×10^5^cells cm^-2^) by gently pulling the cell solution into the upper microchannel using a sterile syringe and needle. After 4 h, a syringe pump was used to perfuse culture medium continuously through the upper microchannel at a constant flow rate (160 μL/h, which produces 0.02 dyne/cm^2^ shear stress) for the first day of culture to make sure that the Caco-2 cells established an intact monolayer, and then the medium flowed at the same rate through both the upper and lower channels thereafter. After Caco-2 cells established an intact monolayer (3 days), cyclic mechanical stretching (1%, 0.15 Hz) is applied to promote further cell differentiation.

### 1.4.2 Transwell culture

We also carried out control studies using static cultures of Caco-2 cells in Transwell plates (Corning Inc., Lowell, MA) containing porous polyester membrane inserts that were pre-coated with the same ECM mixture of type I collagen and Matrigel used in the gut-on-a-chip device. Caco-2 cells also were plated at the same density (3×10^5^ cells /cm^2^) with the medium being refreshed every other day to both the apical and basolateral sides of the Transwell chamber.

## 1.5 Application of Impedance Spectroscopy in TEER Detection

Impedance spectroscopy measurements were carried out daily using an Autolab (PGSTAT302N, Herisau, Switzerland) for 12 days. To reduce the cell damage caused by measuring TEER, in this study, we integrated Ag/AgCl electrodes into the microchannels of gut-on-a-chip. According to the equivalent circuit diagram of impedance measurement, the chip can be divided into four types of components. The first is the resistance of the culture medium inside the channel, and then the porous membrane allows the current to pass vertically and has a vertical resistance. Finally, the cell monolayer can be expressed as the parallel connection of the transmembrane resistance and the cell capacitance. Using the electrochemical impedance spectroscopy of two-electrode mode, firstly, the impedance of the device without cells is measured with 10 μA AC in the frequency range of 1 MHz~10 Hz, and the base resistance is obtained. at the same time, the optimal frequency range (100-10Hz) is selected according to the relationship between frequency and impedance, and the potential difference between readout electrodes is recorded. Gut-on-a-chip was transferred one at a time from the incubator onto an aluminum plate kept at 37 °C to reduce the effect of temperature drift during measurement. Measurements were taken immediately after the transfer. Only approximately 2 minutes were necessary to acquire a full impedance spectrum thus limiting the potential effect of pH changes in the media on the measurements while exposing the chip to ambient pCO2.

## 1.6 Immunofluorescence microscopy

Caco-2 cell monolayer was fixed with 3.7% formaldehyde (MA0192, Meilunbio) in PBS (phosphate-buffered saline, PWL050, Meilunbio) for 15 min washed twice for 5 min with PBS and permeabilized with 0.3% Triton X-100 (Cat#T8002, Solarbio) in PBS for 10 min. After washing with 4% FCS in PBS, cells were incubated with blocking solution (2% FCS, 2% bovine serum albumin (BSA) (A8010, Solarbio), 0,1% Tween 20 (Sigma#P9416) in PBS) for 45 min. Subsequently, cells were incubated with primary antibodies for 60 min or at 4 °C overnight, washed three times, incubated with secondary antibodies for 30 min and washed three times with 4% FCS in PBS. The following antibodies were used for immunohistochemistry: Rabbit a-ZO-1 (ab221547, Abcam, dilution 1:200), Mouse a-Ezrin (ab40839, Abcam, dilution 1:125), Rabbit a-Piezo1(28511-1-AP, Proteintech, dilution 1:200), Mouse a-DMT1 (sc-166884, Santa, 1:200), Donkey a-Rabbit AlexaFluor 647 (ab150075, Abcam, 1:1000), Goat a-Mouse AlexaFluor 488 (150113, Abcam, 1:1000). All steps were performed at room temperature (RT). Cells were co-stained with DAPI (10 μg/ml, meilunbio). Microscopy was performed with laser scanning confocal microscopy (Zeiss LSM880). Quantification of the immunofluorescence images was performed using ImageJ software based on the mean fluorescence intensity on a per-cell basis.

## 1.7 Optimization of the reduction time of AuNPs

To further improve the electrochemical performance of the electrode, the reduction time of AuNPs was optimized. Six electrodes were prepared by the different reduction time lengths ranging from 0s to 200 s. The oxidation peak current was increased by the reduction time of AuNPs and trends to be plateaued after 175s. This is because the electrode surface was fully saturated by a sufficient amount of AuNPs, more AuNPs trended to accumulate on the electrode surface and cannot increase the active sites. Therefore, the oxidation peak current does not change much after 175s, so 175s was chosen as the best reduction time for AuNPs.

## 1.8 Effective elimination of microbubbles in chips

In the process of infusing the culture medium into the gut-on-a-chip, air bubbles are easy to appear in the microchannel. The existence of bubbles will seriously affect the growth state of cells, and it is not easy to expel. There are two reasons for the formation of bubbles, one is the temperature difference between the incubator and the outside. When the culture medium flows into the incubator, the oxygen dissolved in the culture medium overflows and forms tiny bubbles because of the temperature difference. Second, the seal of the device is not strict, and air enters the device to form bubbles. Most of the reasons are caused by the first situation. For bubble elimination, most of the literature shows the use of bubble remover to eliminate bubbles, but the cost of this method is high. In this paper, we adopt a simple and convenient way to eliminate bubbles. First of all, to eliminate the effect of temperature difference, the culture medium is heated in a water bath and heated to 37 ℃, which is equal to the temperature of the incubator, so that the dissolved air in the culture medium can be released in advance. Then, a liquid storage bottle is added in the middle of the perfusion pipe. The liquid storage bottle contains the culture medium at 37 ℃ and is placed in the incubator all the time. Outside the incubator, the pressure and fresh culture medium are provided by using an injection pump, and the culture fluid in the bottle can be transported to the gut-on-a-chip because of the pressure difference in the storage bottle. In this way, not only the existence of air bubbles can be eliminated effectively, but also the culture medium can be changed conveniently and quickly.

# 2. Supporting Figures


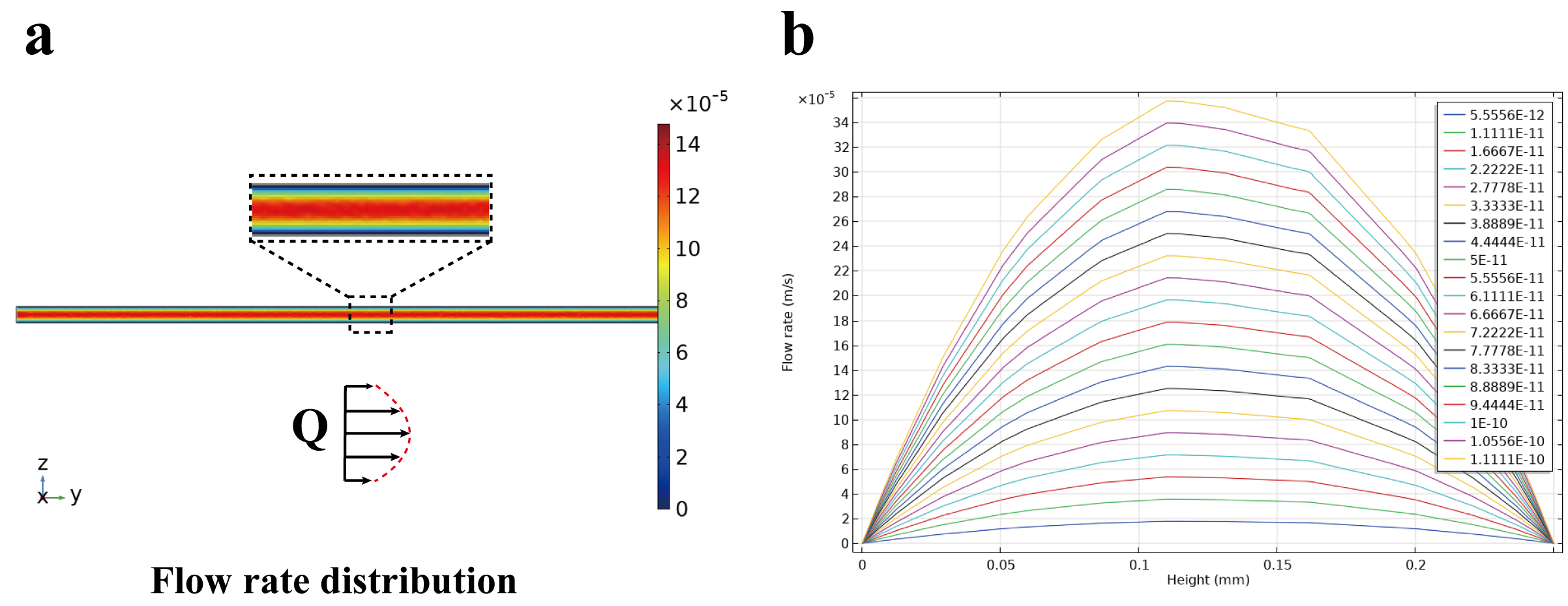


**Figure.S1 Simulation of flow Field in channel of gut-on-a-chip. a),** Finite element analysis of flow field in microchannel. **b),** The gut-on-a-chip has the flow field distribution under different flow rate culture medium, in which the x-axis represents the height of the upper channel.


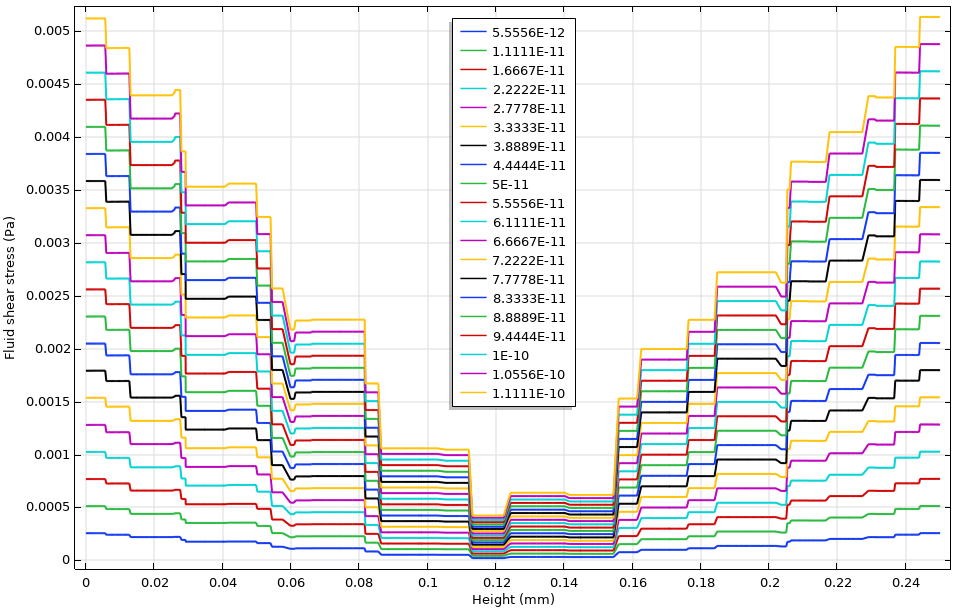


**Figure.S2 The distribution of wall shear stress caused by different flow velocity of culture medium in microchannel.** When the velocity is 4.44×10^-11^m/s, the shear stress of 0.002Pa (0.02dyne/cm^2^) is produced.


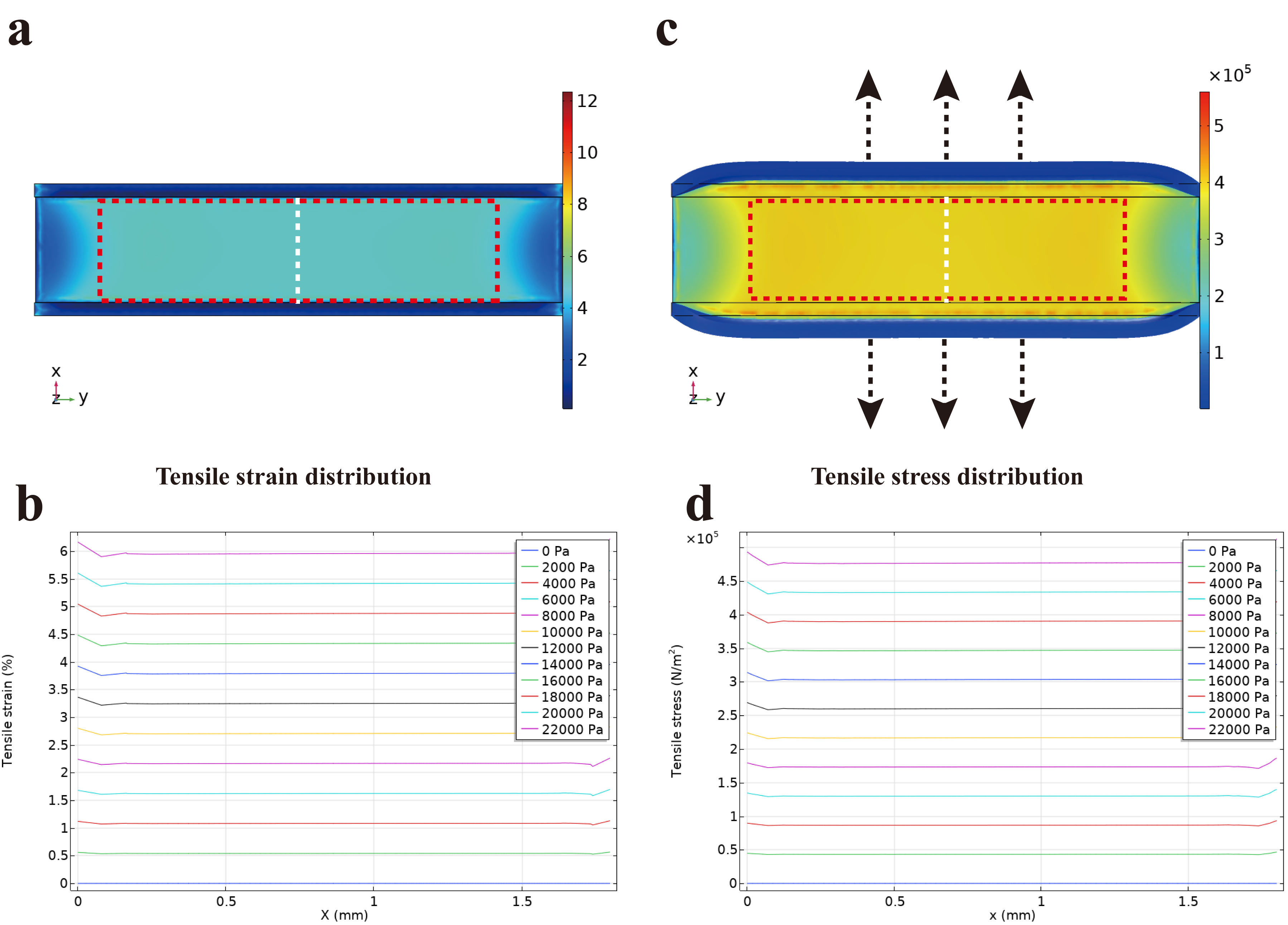


**Figure.S3 Strain and Stress distribution.** **a),** FEA of tensile strain distribution of porous membrane. **b),** Under the same mechanical stretching, the strain distribution along the X-axis (white dotted line) had no significant fluctuation. **c),** FEA of tensile stress of porous membrane. **d),** Under the same mechanical stretching, the stress distribution along the X-axis (white dotted line) had no significant fluctuation.


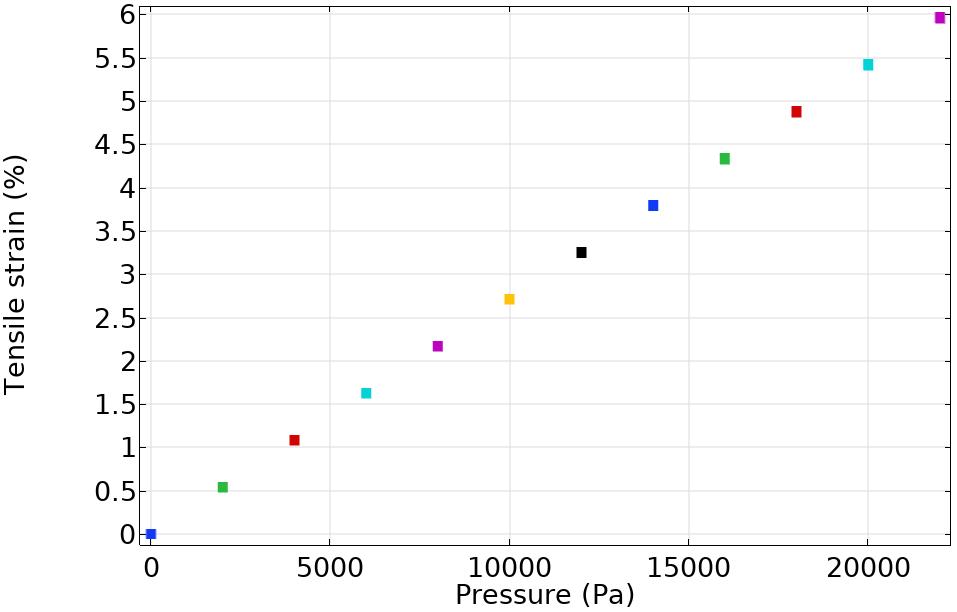


**Figure.S4 Variation of surface strain of porous membrane under different pressure tension.** As the inspiratory pressure increases from 0 to 20 kpa, the strain increases linearly from 0 to 5.4%.


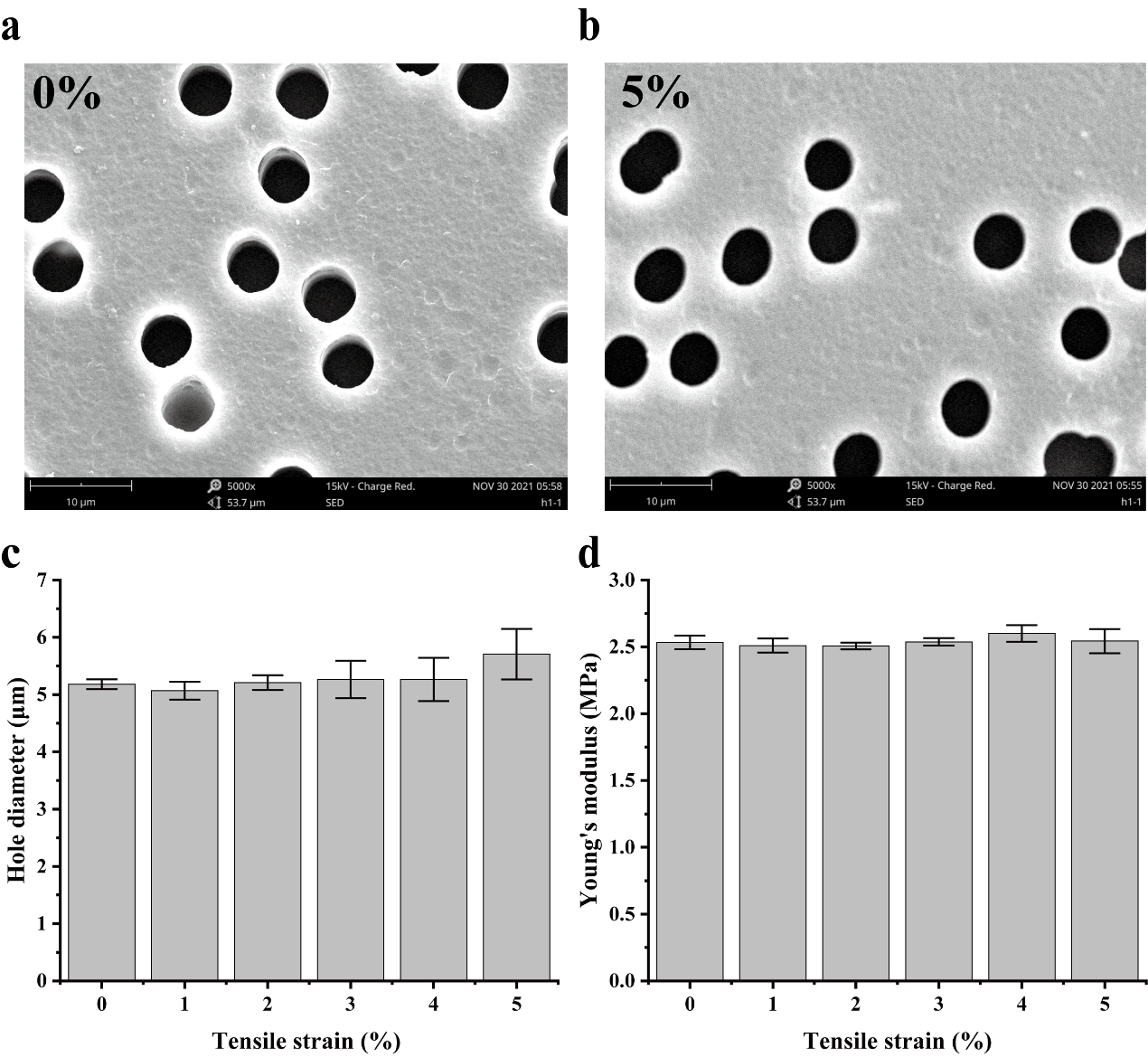


**Figure.S5 Variation of pore size and Young's Modulus of the porous membrane. a),** SEM image of the unstretched porous membrane. **b),** Under 5% strain, the SEM image of the porous membrane was stretched continuously for 10 days. **c),** The change of pore diameter of the porous membrane after stretching for 10 days with different tensile strain (0-5%) (n=3). **d),** The change of Young's modulus of the porous membrane after stretching for 10 days with different tensile strain (0-5%) (n= 3).


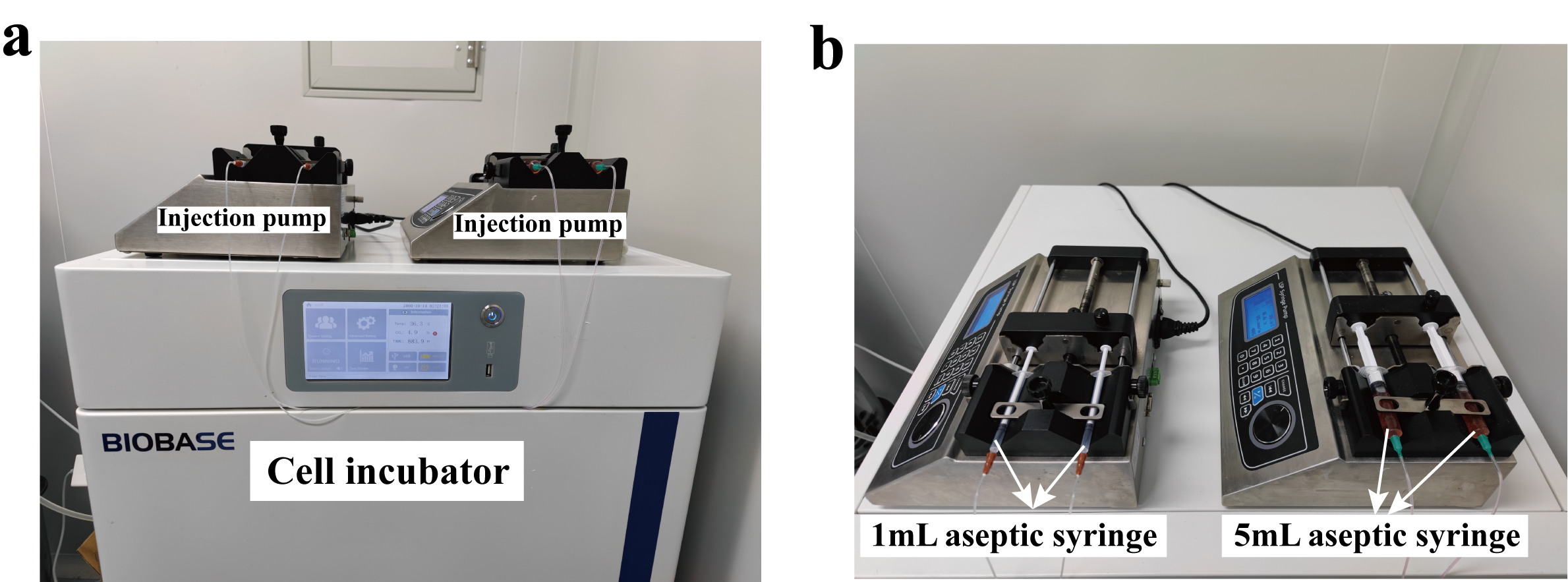


**Figure.S6 The whole culture system.**

**
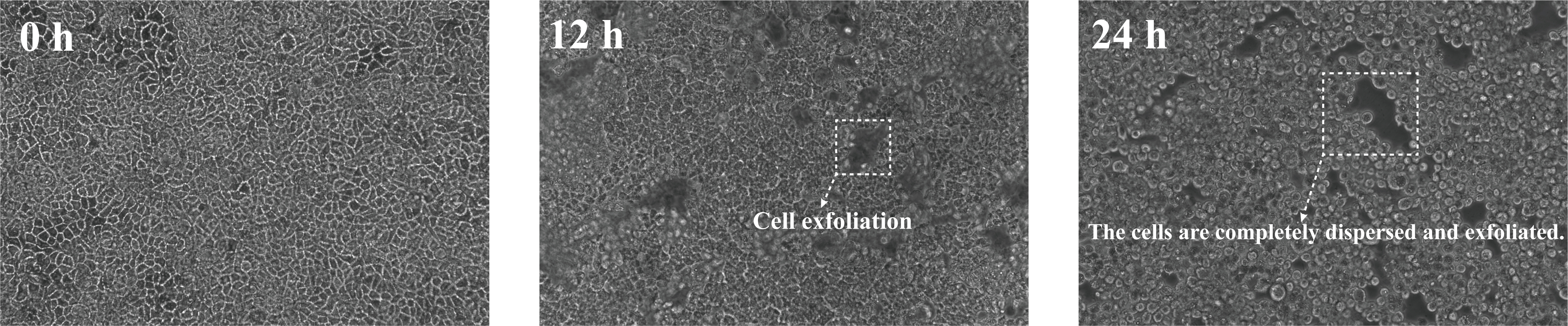
**

**Figure.S7** The change of cell morphology with time after adding 100 μ M Hg(Ⅱ) to the monolayer in static culture. Hg(Ⅱ) can destroy the tight junctions between cells, caused cells to exfoliation and die.


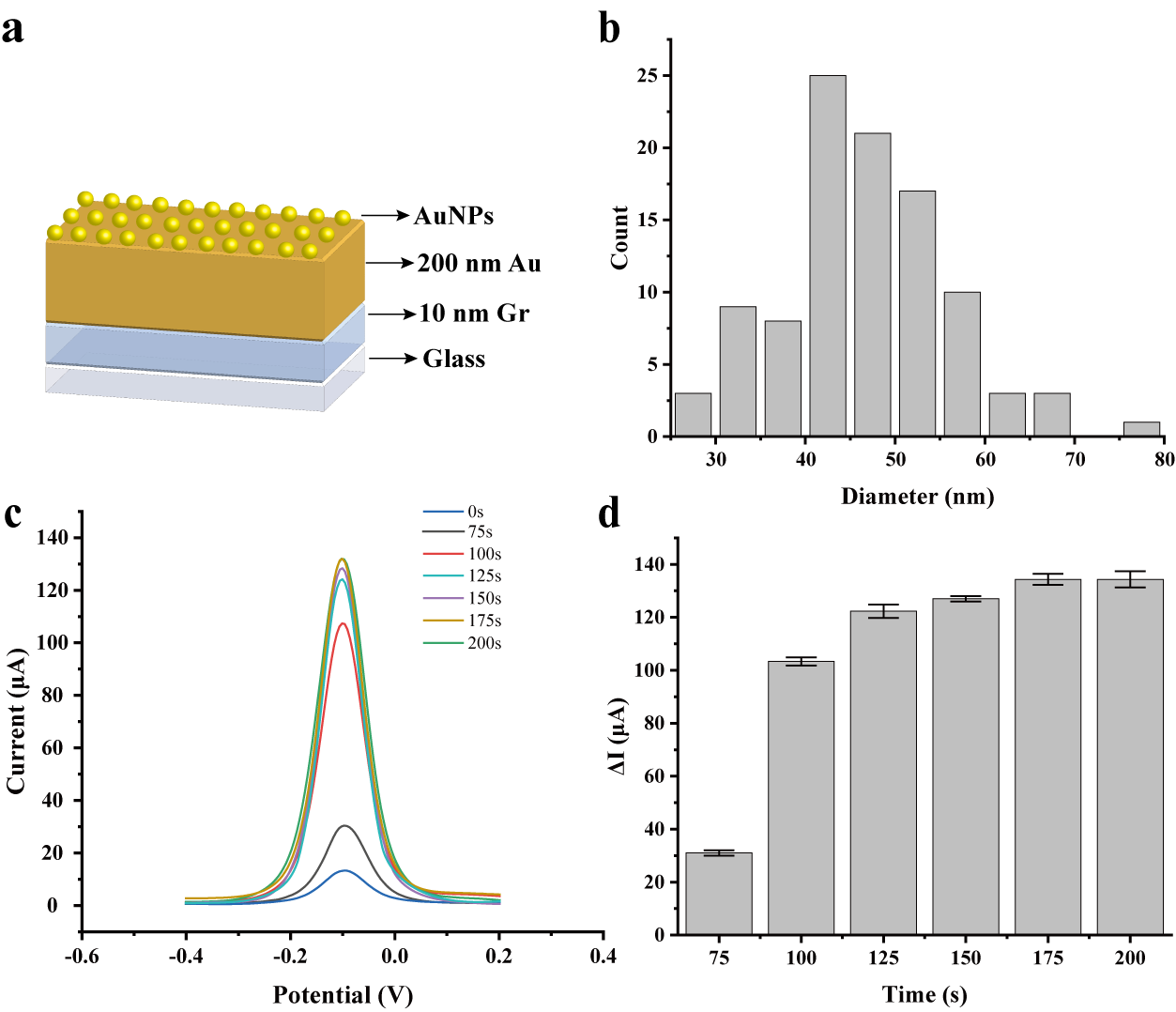


**Figure.S8 AuNPs Optimization of reduction time. a),** Schematic diagram of electrode material. **b,)** The particle size distribution of AuNPs. The particle size is mainly distributed in the range of 400-500nm. **c, d),** The AuNPs reduction time is optimized, and the reduction time tends to be saturated after 175s (n=3).


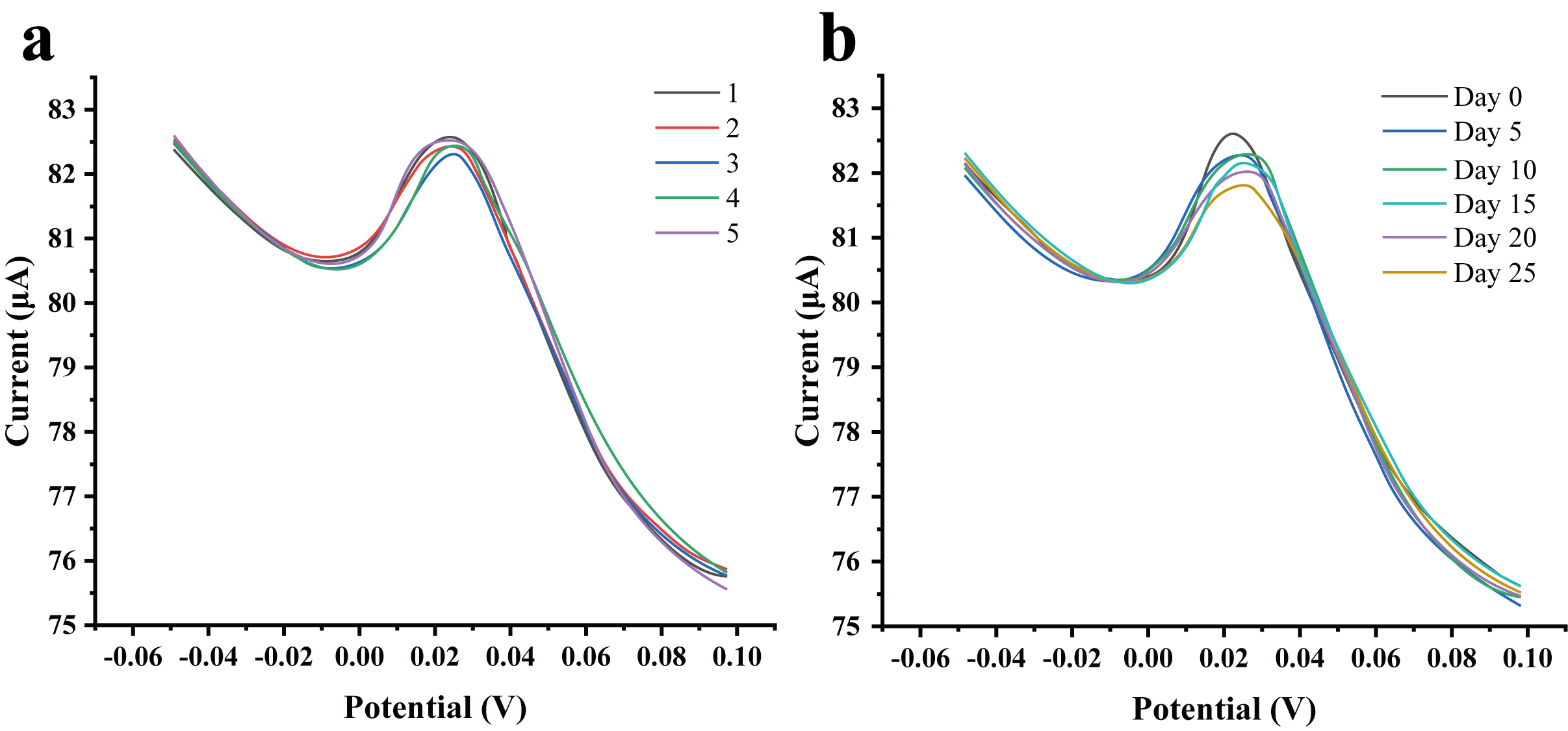


**Figure.S9 Verification of electrode consistency and stability. a, b),** Electrode consistency verification, five electrodes were randomly selected to detect the DPV response of 50 nM Hg(Ⅱ), the relative standard deviation was 4%, and the electrode had good consistency (n=3). **c, d),** The stability of the electrode was verified. The DPV response of 50 nM Hg(Ⅱ) was detected every five days. During the 25-day detection process, the peak current decreased slightly with the increase of time, and the peak current on the 25th day was 86% of the initial value. The electrode has good stability (n=3).

**
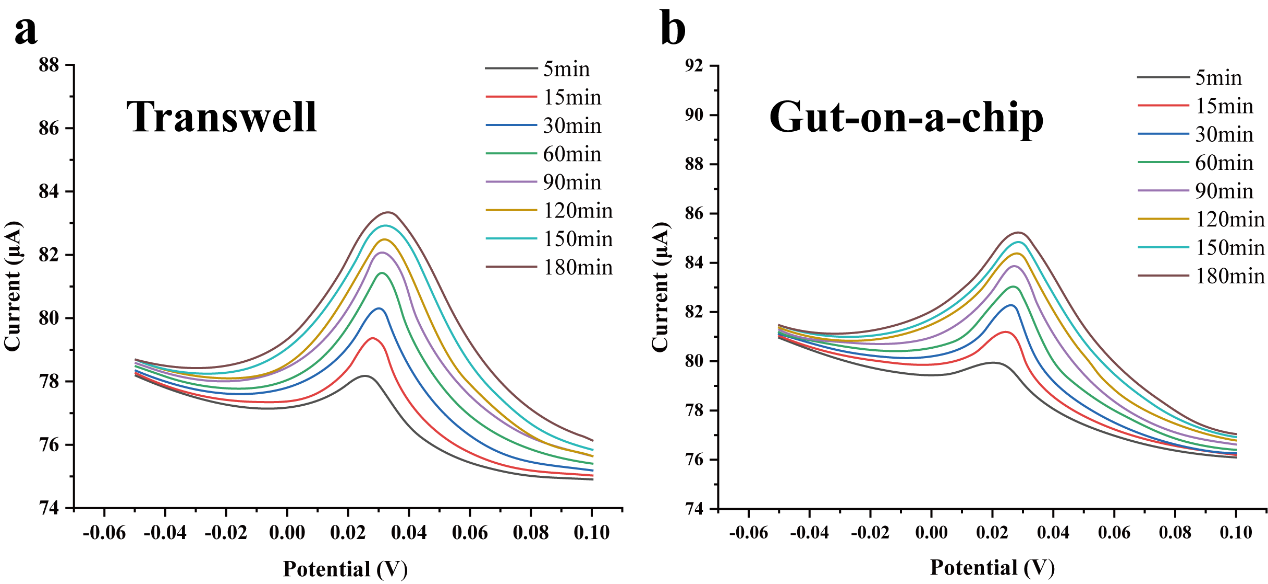
**

**Figure.S10 Hg(Ⅱ) absorption detection. a),** DPV response of Transwell statically cultured cells to mercury ion uptake by monolayer. **b),** The DPV response of the monolayer of cells to absorb mercury ions was dynamically cultured by Gut-on-a-chip.


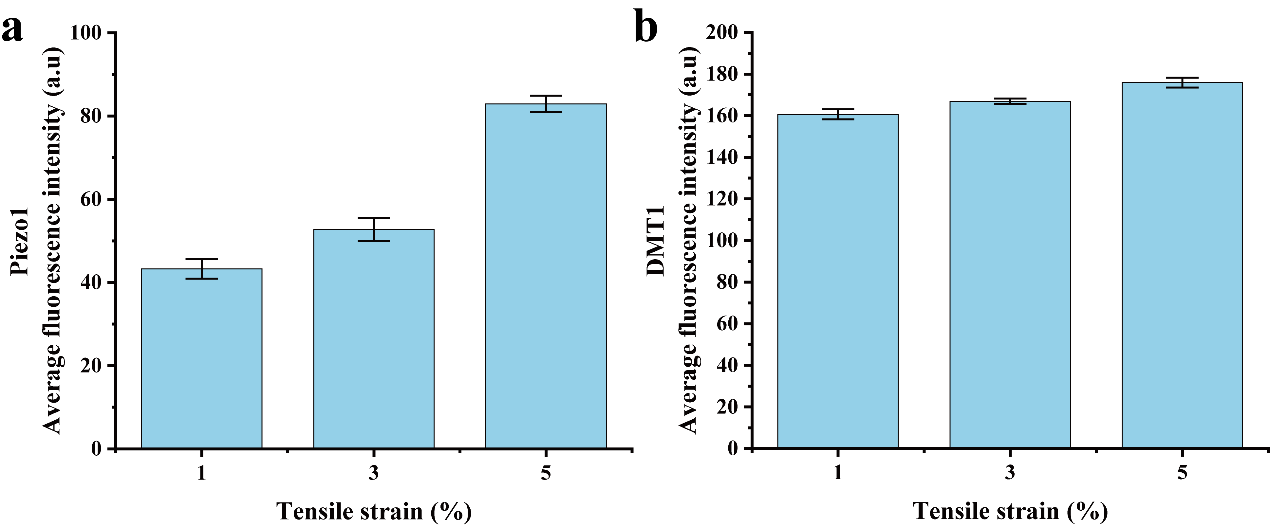


**Figure.S11 Average fluorescence intensity. a),** Changes of average fluorescence intensity of Piezo1 protein under different mechanical stretching (n=3). **b),** Changes of average fluorescence intensity of DMT1 protein under different mechanical stretching (n=3).

**
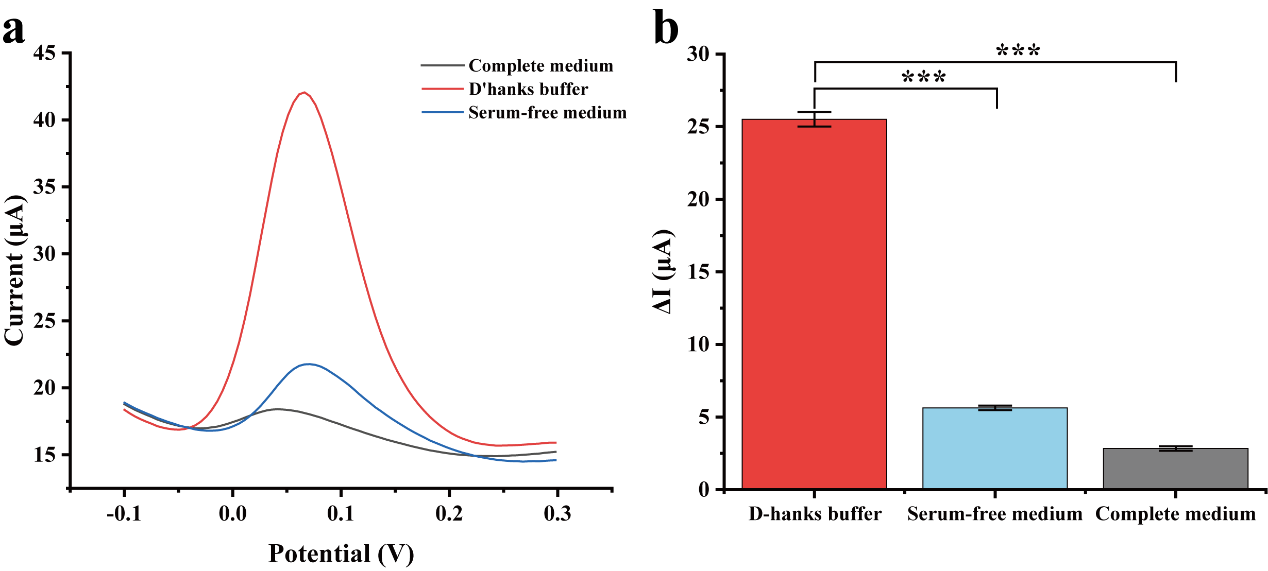
**

**Figure.S12** **The effect of culture medium on Hg(Ⅱ) detection.** **a),** DPV response of Hg(Ⅱ) in three different solutions. **b),** Comparison of current difference of Hg(Ⅱ) in three kinds of solution.

Hg(Ⅱ) were diluted to the same concentration using D-hanks buffer, (DMEM + 1% penicillin/streptomycin) and complete medium (DMEM + 1% penicillin/streptomycin + 10% FBS), and their DPV responses were detected by electrochemical workstation. The results showed that the culture medium had a significant effect on the detection of Hg(Ⅱ)(n=3, ***P<0.001), and the Hg(Ⅱ) in D-hanks buffer had a higher DPV response. The low peak value of Hg(Ⅱ) current in the culture medium may be due to the influence of organic molecules in the culture medium. At the same time, it was found in the experiment that the culture medium has a very high resistance, which may also be the reason for the decrease of peak current.

Although the D-hanks buffer can get a higher peak current, we found that the cell monolayer dispersed when the cell monolayer was exposed to the D-hanks buffer for more than three hours. Therefore, to ensure the integrity of the cell barrier and good detection effect, in the later absorption experiment, we added a serum-free culture medium containing 10 μM Hg(Ⅱ) in the upper channel and D-hanks buffer without Hg(Ⅱ) in the lower channel.


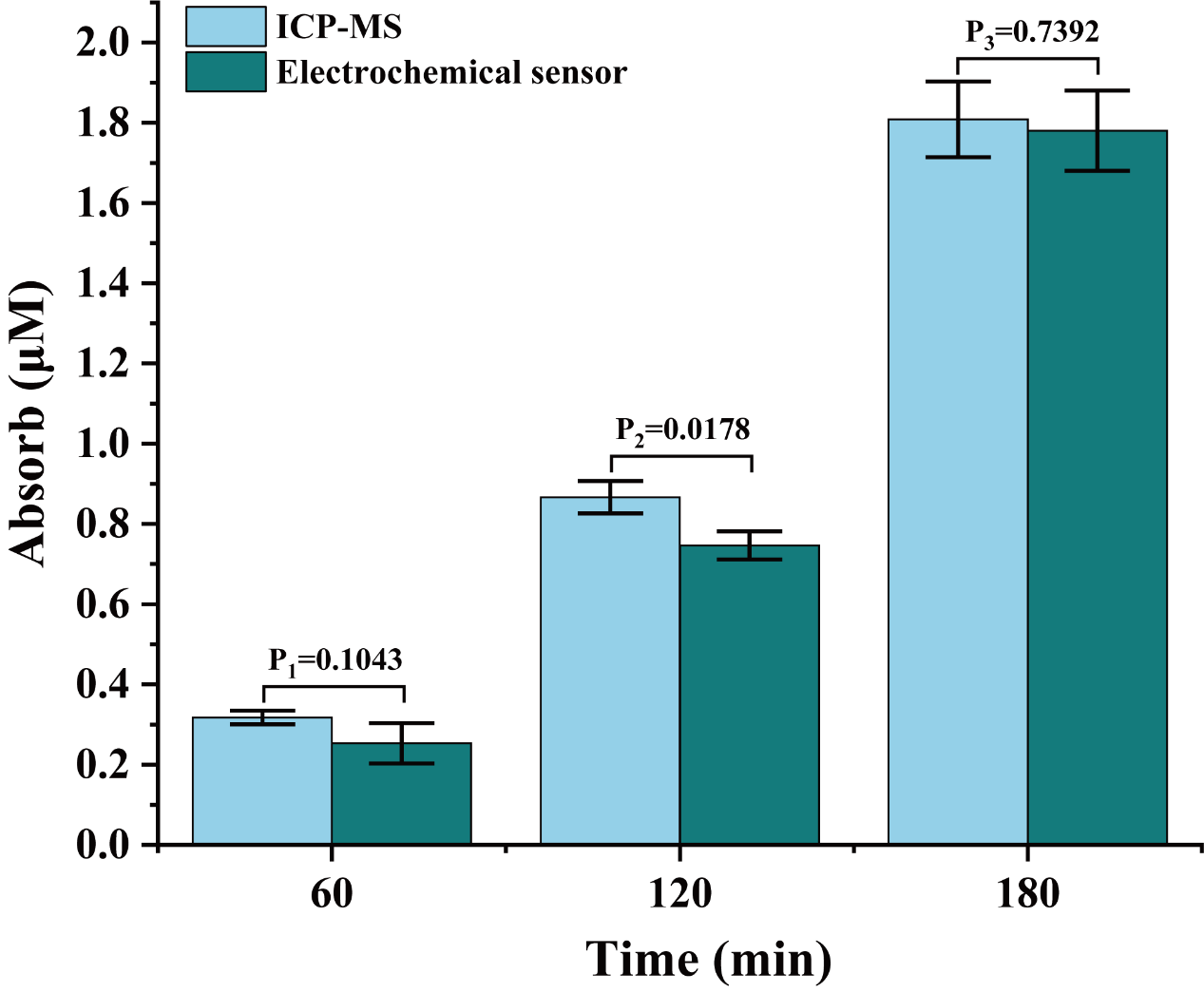


**Figure.S13** **Performance comparison between ICP-MS and electrochemical sensor**

To determine the accuracy of the electrochemical sensor made in this study to detect Hg(Ⅱ), we used an electrochemical sensor and ICP-MS to detect the Hg(Ⅱ) absorbed by intestinal epithelial cells, and compared the two results. Comparison of electrochemical sensor and ICP-MS for detection of Hg(Ⅱ). The corresponding P values were P_1_ = 0.1043, P_2_ = 0.0178 and P_3_ = 0.7392, respectively (n=3).
